# Open axillary approach alternative access for stenting of external iliac total occlusion

**DOI:** 10.1016/j.radcr.2022.03.022

**Published:** 2022-04-07

**Authors:** Karthigesu Aimanan, Putera Mas Pian, Kumaraguru V K Pillay, Firdaus Hayati, Hanif Hussein

**Affiliations:** a Department of Surgery, Kuala Lumpur Hospital, Kuala Lumpur, Malaysia; b Department of Surgery, Faculty of Medicine and Health Sciences, Universiti Malaysia Sabah, Kota Kinabalu, Sabah, Malaysia

**Keywords:** Axillary artery approach, Aortoiliac occlusion, External iliac stenting

## Abstract

A primary endovascular approach is the mainstay of intervention for type C aortoiliac disease. When the femoral artery is unsuitable, upper extremity access can be critical in the setting of severe tortuosity or occlusive disease. The axillary artery represents alternative upper extremity access that may accommodate larger sheath sizes for therapeutic interventions. A 44-year-old gentleman with a history of right below-knee amputation was referred to the vascular unit with a left foot non-healing wound post wound debridement for diabetic foot ulcer. On examination, the left foot was non-salvageable with pitting oedema extended until knee level. Left lower limb pulses were non-palpable from femoral downwards. A biphasic signal was audible at the left femoral and monophasic at the popliteal. Photoplethysmography showed poor flow distally. Computed tomography angiogram revealed a 12 cm long segment total occlusion of the left external iliac artery just below the bifurcation of iliac vessel. On the right side, there was a long segment occlusion of the superficial femoral artery and calcified common femoral artery. The left axillary artery was used as an access and angioplasty was performed successfully. The advantages of upper extremity access in the axillary artery include the relatively large size and lower atherosclerotic burden. Larger profile stents for aortoiliac occlusion can easily be handled with a good strength through an axillary approach which is antegrade compared to a retrograde femoral approach. With the advancement of safety features of endovascular devices complications with an axillary approach have become less in the recent era.

## Introduction

Femoral artery remains the vascular access site of choice for endovascular procedures requiring large-bore arterial access including endovascular aortic aneurysm repairs (EVAR) and peripheral arterial diseases (PAD). It is common to encounter the challenge of treating structural or complex anatomy in patients with significant concomitant PAD. The contralateral femoral artery has been the original access site when treating external iliac occlusion via percutaneous endovascular intervention [1]. Axillary vascular access has recently re-emerged as an alternative access option for large bore arteriotomies in patients with aortic aneurysm or coronary interventions. Yet, the existing literature on trans-axillary interventions for long-segment iliac occlusion is scarce. We report a 44-year-old gentleman with a left non-healing wound ulcer who underwent trans-axillary interventions for long-segment iliac occlusion and discuss its management plans.

## Case report

A 44-year-old gentleman with a previous history of right below-knee amputation (BKA) was referred to the vascular unit with a left foot non-healing wound post wound debridement for diabetic foot ulcer. Previously he was ambulating with a prosthetic leg on the right BKA stump. On examination, the left foot was non-salvageable with pitting oedema extended until knee level. Left lower limb pulses were non-palpable from femoral downwards. A biphasic signal was audible at the left femoral and monophasic at the popliteal. Photoplethysmography showed poor flow distally. On the right side, the femoral pulse was palpable but the popliteal pulse was non-palpable with an audible biphasic doppler signal. There was no wound on the right BKA stump and the patient has no complaints on the right side. Our impression was left critical limb ischemia secondary to aortoiliac disease. We proceeded with computed tomography (CT) angiogram to delineate the vessels given normal renal function.

**Fig. 1:**
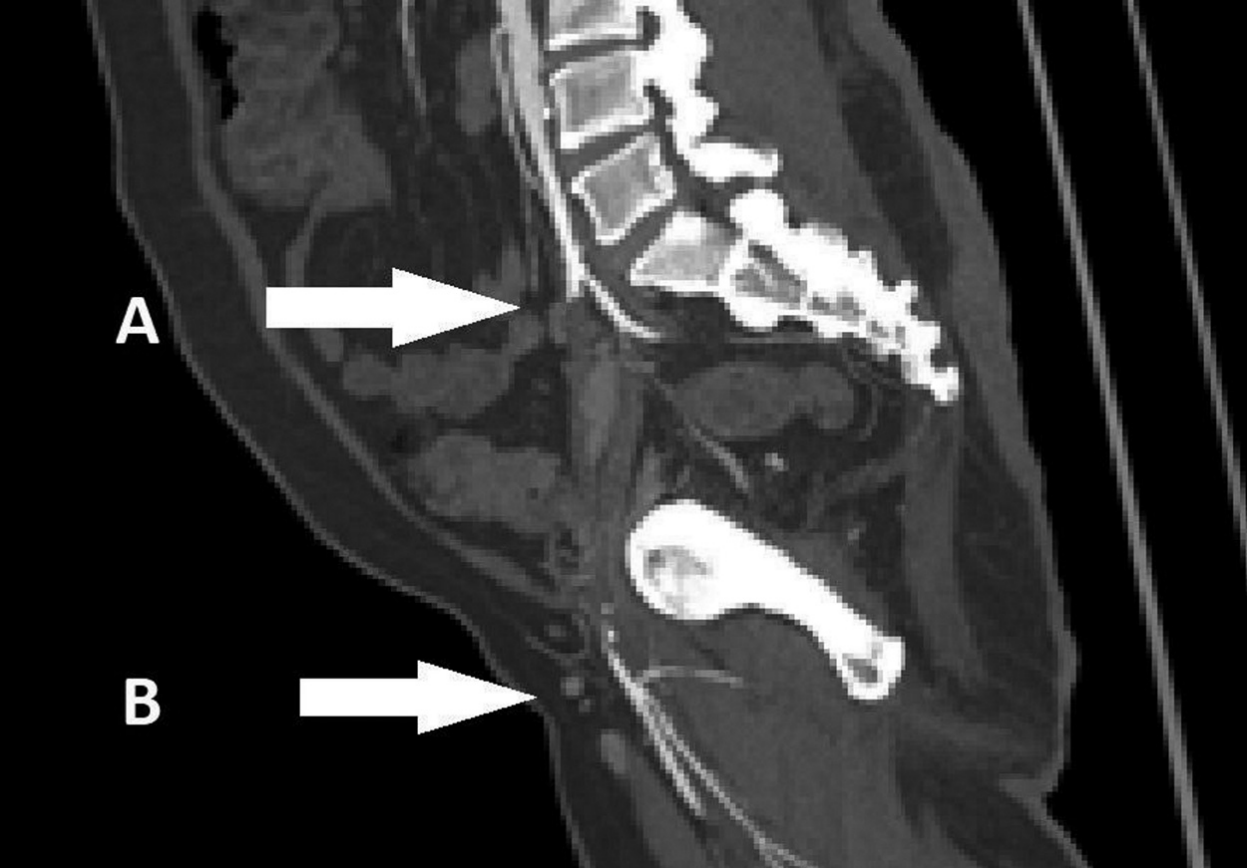
Long segment occlusion from the origin of left CIA (A) until the proximal CFA (B).

CT angiogram revealed a 12 cm long segment total occlusion of the left external iliac artery (EIA) just below the bifurcation of iliac extending until proximal common femoral artery (CFA) (Fig. 1). On the right side, there was a long segment occlusion of the superficial femoral artery and calcified common femoral artery. These findings on the right side made us unable to use the contralateral retrograde puncture approach which might compromise the blood supply distally. We have explained to the patient the need for left above-knee amputation (AKA) due to the extent of infection and the need for revascularization to maintain the AKA stump viability. We chose 2 self-expanding covered stents with a diameter of 6 mm each, with a profile of 8Fr. His brachial artery on the left upper limb was small with a diameter of 3 mm, which made the access not possible. The left axillary artery was used instead for the purpose of access with a backup plan of converting into an axillo-femoral bypass if the stent failed.

**Fig. 2:**
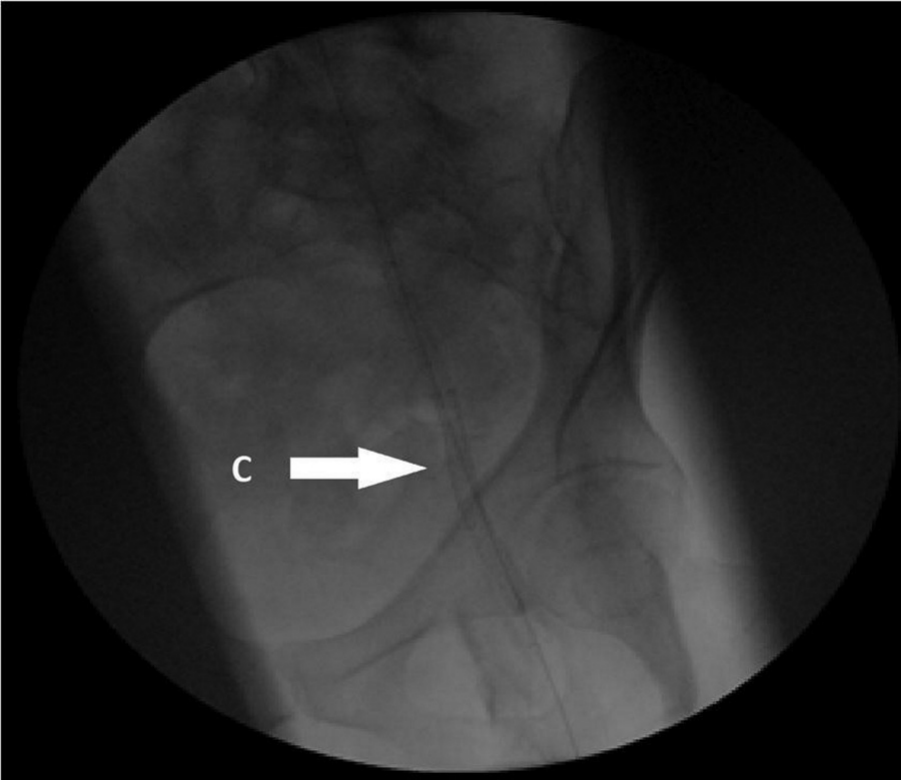
Two self-expanding covered stents (C) deployed from the origin of left EIA until proximal CFA.

Axillary artery cut down was performed and an 80 cm x 8 Fr supporting catheter was inserted. Angio run was consistent with the CT angiogram finding, which showed a long segment occlusion of EIA. Complete total occlusion wire 0018 30 g, was successfully used to bypass the occlusion into CFA. Vessel preparation was performed with a 6 mm PTA ballon prior to stenting. Two self-expanding stents 80 mm x 6 mm were used with an overlap of 2 cm to stent the EIA (Fig. 2). Angio run post stenting shows flow into profunda and superficial femoral artery distally. Subsequently, an above-knee amputation was performed for the patient. Postoperatively the patient's femoral pulse on the left side was palpable and the wound over his AKA stump was well healed. He is under our follow up for the past 6 months and had no new limb related events or revascularization.

## Discussion

EIA occlusions are challenging due to access difficulty. The contralateral femoral artery is the original access site for percutaneous endovascular intervention in external iliac occlusion [1]. In patients with a calcified or occluded contralateral femoral artery, upper extremity access has become increasingly common to facilitate parallel grafting. The advantages of upper extremity access in the axillary artery include the relatively large size and lower atherosclerotic burden. In addition, this approach avoids hostile iliac anatomy and challenging femoral access due to obesity or inguinal scarring and infection. An antegrade approach offers a more favorable angle to the target vessel and avoids difficulty in crossing over an acute-angled bifurcation by a retrograde femoral approach [2].

Device selection plays an important role in deciding between the brachial or axillary approach. A self-expandable covered stent is our choice in this patient due to its superiority in terms of flexibility and can also be used as a bailout stent in the event of an external iliac perforation. These stents usually come in a larger profile (8 Fr) which necessitates at least a vessel size of more than 3mm in diameter to avoid complications. An open axillary approach also avoids the use of a longer sheath or longer balloon compared to the brachial artery approach. However, if a plan is to intervene in a distal disease such as in the leg this advantage will become void. Besides that, an axillary approach in an external iliac occlusion also facilitates alternative backup surgery such as axillo-femoral bypass in the event of unsuccessful stenting.

Limitations of the axillary approach compared to a brachial artery are nerve injury and high-risk ischemia due to its proximal anatomy. The rate of complications in early studies was high, but this was in the era of larger angiographic catheters [3,4]. The use of large (7 and 8 Fr) angioplasty catheters, without a sheath to protect the artery at the time of removal of the balloon catheter, seemed particularly likely to cause complications [4]. With the advancement of devices with delivery sheath, these complications have become less in the recent era [5].

The percutaneous technique is a known alternative method to get into trans axillary access. Schäfer et al. were among the first to describe the technique of percutaneous trans-axillary artery access for TAVR in a large case series format [6]. The advantage of this technique includes, among other aspects, potential compressibility of the vessel against the second rib for manual hemostasis, if needed. As such, Schäfer et al. observed a significantly lower complication rate than was reported in the previous studies [7]. Despite a reported lower complication rate, open access is preferable in our center for axillary to better visualize the nerves and avoid them completely during dissection. Due diligence in establishing hemostasis upon closure is vital to minimize hematoma and surgical site infection in the open approach.

## Conclusion

The advantages of upper extremity access in the axillary artery include the relatively large size and lower atherosclerotic burden. Larger profile stents for aortoiliac occlusion can easily be handled with a good strength through an axillary approach which is antegrade compared to a retrograde femoral approach. With the advancement of safety features of endovascular devices complications with an axillary approach have become less in the recent era.

## Patient consent

An informed consent for publication was obtained from the patient and it is available upon request.
